# Genetic Deficiency of Indoleamine 2,3-dioxygenase Aggravates Vascular but Not Liver Disease in a Nonalcoholic Steatohepatitis and Atherosclerosis Comorbidity Model

**DOI:** 10.3390/ijms23095203

**Published:** 2022-05-06

**Authors:** Aastha Arora, Gustavo Luis Tripodi, Ilona Kareinen, Martin Berg, Maria Josefa Forteza, Anton Gisterå, Silke Griepke, Felipe Beccaria Casagrande, Joilson O. Martins, Dulcineia Saes Parra Abdalla, Jennifer Cole, Claudia Monaco, Daniel F. J. Ketelhuth

**Affiliations:** 1 Division of Cardiovascular Medicine, Center for Molecular Medicine, Department of Medicine, Karolinska Institute, Karolinska University Hospital, 17164 Stockholm, Sweden; gustavolt.90@gmail.com (G.L.T.); ilona.kareinen@gmail.com (I.K.); martin.berg@skane.se (M.B.); maria.forteza.de.los.reyes@ki.se (M.J.F.); anton.gistera@ki.se (A.G.); 2 Department of Cardiovascular and Renal Research, Institute of Molecular Medicine, University of Southern Denmark, 5000 Odense-C, Denmark; arora@health.sdu.dk (A.A.); sgnielsen@health.sdu.dk (S.G.); 3 Department of Clinical and Toxicological Analyses, School of Pharmaceutical Sciences, University of Sao Paulo, Sao Paulo 05508-000, Brazil; felipe.casagrande@ki.se (F.B.C.); martinsj@usp.br (J.O.M.); dspa@usp.br (D.S.P.A.); 4 Kennedy Institute of Rheumatology, University of Oxford, OX3 7FY Oxford, UK; jennifer.cole@kennedy.ox.ac.uk (J.C.); claudia.monaco@kennedy.ox.ac.uk (C.M.)

**Keywords:** IDO, inflammation, atherosclerosis, NASH, immunometabolism

## Abstract

Nonalcoholic steatohepatitis (NASH) is a chronic liver disease that increases cardiovascular disease risk. Indoleamine 2,3-dioxygenase-1 (IDO1)-mediated tryptophan (Trp) metabolism has been proposed to play an immunomodulatory role in several diseases. The potential of IDO1 to be a link between NASH and cardiovascular disease has never been investigated. Using *Apoe^−/−^* and *Apoe^−/−^Ido1^−/−^* mice that were fed a high-fat, high-cholesterol diet (HFCD) to simultaneously induce NASH and atherosclerosis, we found that *Ido1* deficiency significantly accelerated atherosclerosis after 7 weeks. Surprisingly, *Apoe^−/−^Ido1^−/−^* mice did not present a more aggressive NASH phenotype, including hepatic lipid deposition, release of liver enzymes, and histopathological parameters. As expected, a lower L-kynurenine/Trp (Kyn/Trp) ratio was found in the plasma and arteries of *Apoe^−/−^Ido1^−/−^* mice compared to controls. However, no difference in the hepatic Kyn/Trp ratio was found between the groups. Hepatic transcript analyses revealed that HFCD induced a temporal increase in tryptophan 2,3-dioxygenase (*Tdo2)* mRNA, indicating an alternative manner to maintain Trp degradation during NASH development in both *Apoe*^−/−^ and *Apoe*^−/−^*Ido1*^−/^mice^−^. Using HepG2 hepatoma cell and THP1 macrophage cultures, we found that iron, TDO2, and Trp degradation may act as important mediators of cross-communication between hepatocytes and macrophages regulating liver inflammation. In conclusion, we show that *Ido1* deficiency aggravates atherosclerosis, but not liver disease, in a newly established NASH and atherosclerosis comorbidity model. Our data indicate that the overexpression of *TDO2* is an important mechanism that helps in balancing the kynurenine pathway and inflammation in the liver, but not in the artery wall, which likely determined disease outcome in these two target tissues.

## 1. Introduction

Atherosclerosis is the underlying cause of most cardiovascular diseases (CVDs) and the leading cause of morbidity and mortality worldwide [1,2]. Atherosclerosis development is influenced by several risk factors, including dyslipidaemia, hypertension, smoking, and diabetes; targeting these risk factors is currently the main strategy to combat the CVD burden. Despite all the recent developments in medicine, only up to one-third of major clinical consequences of atherosclerosis, e.g., myocardial infarction, seem to be prevented by using the current guidelines [3].

It is now well recognized that atherosclerosis is a chronic inflammatory disease that is likely initiated by the accumulation of low-density lipoprotein (LDL) in the artery wall. The accumulation and modifications of LDL trigger maladaptive innate and adaptive immune responses in the artery wall, driving the formation of an atherosclerotic plaque, as well as a fibrous cap that, upon rupture, can cause thrombosis and a CVD event [4]. Large preclinical and, more recently, clinical evidence indicate that immunomodulation of vascular inflammation could be used to reduce the CVD burden beyond current guidelines for handling classic disease risk factors [5].

Activation of the immune system is not only well established within the pathophysiology of atherosclerosis, but also plays a major role in the development of nonalcoholic fatty liver disease (NAFLD), which can progress to nonalcoholic steatohepatitis (NASH); the latter that is defined as the combination of liver steatosis, parenchymal damage (hepatocyte apoptosis and ballooning, and focal necrosis), lobular and/or portal inflammation, and variable degrees of fibrosis [6,7]. Due to its association with obesity, type 2 diabetes, and the ectopic accumulation of lipids in the liver parenchyma, NASH has been considered an advanced hepatic component of metabolic syndrome and an additional risk factor for CVD [7].

Indoleamine 2,3-dioxygenase-1 (IDO1), the rate-limiting enzyme of the kynurenine pathway of tryptophan (Trp) metabolism, has been identified as a key immunomodulatory enzyme implicated in different diseases, including atherosclerosis and liver disease. It has been proposed that the local depletion of Trp and/or the production of potent bioactive metabolites of this pathway, collectively known as kynurenines, can modulate immune cell functions such as activation, proliferation, and migration [8].

While increased IDO1 expression, in the great majority of cases, has been implicated in atheroprotection [8,9,10,11,12,13,14,15,16], its role in liver inflammation and NAFLD/NASH is less clear. It has been shown that, upon high-fat diet feeding or the injection of CCL4, *Ido1*-deficient mice present increased hepatic inflammation and fibrosis [17,18]. IDO-dependent regulation of IL-17 release has been suggested as a major mechanism attenuating liver fibrosis [19,20]. However, IDO expression has also been associated with obesity and worsening insulin resistance through the regulation of intestinal permeability, which also influences liver steatosis [21].

Although NAFLD/NASH-related mortality is usually linked to adverse hepatic outcomes such as cirrhosis, liver failure, and hepatocellular carcinoma, CVDs are the main cause of mortality among patients with liver disease [22,23]. Thus, it has been hypothesized that NAFLD/NASH-related steatosis is a chronic inducer of low-grade hepatic inflammation and the source of several immunomodulatory mediators, which, when systemically released, could accelerate vascular inflammation and the development of CVDs [24]. Yet, the precise molecular mechanisms underlying the relationship between NASH and atherosclerosis remain unclear.

In this study, we validated a new murine model that develops both NASH and atherosclerosis. Using *Apoe^−/−^* and *Apoe^−/−^Ido1^−/−^* mice that were fed a high-fat and cholesterol-rich diet, we demonstrate an essential role of IDO1 in accelerating vascular but not liver disease in the same animals. Our results indicate that disease-related factors promote the upregulation of tryptophan 2,3-dioxygenase (*Tdo2*) in the liver, which could help maintain local Trp degradation and prevent the aggravation of NASH.

## 2. Results

### 2.1. Apoe^−/−^ Is a Suitable Strain for Studying Atherosclerosis and NASH as Comorbidities—Model Validation

**Figure 1:**
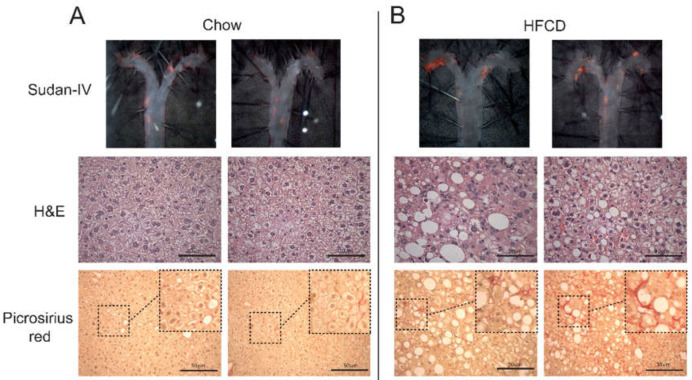
Characterization of the NASH and atherosclerosis dual model. *Apoe^−/−^* mice were fed chow or a high-fat-cholesterol diet (HFCD) for 7 weeks, and representative pictures of atherosclerotic disease and NASH are shown; complete descriptive data of chow- and HFCD-fed mice are shown in Table 1. Top panels show *en face* lipid staining with Sudan-IV; middle panels show H&E staining showing hepatocyte ballooning; and the bottom panels show collagen deposition detected by picrosirius red staining in (**A**) chow- and (**B**) HFCD-fed *Apoe^−/−^* mice. Bar = 50 µm.

Both male and female *Apoe^−/−^* mice were fed HFCD for 3.5 and 7.0 weeks, and systemic organ-specific changes were evaluated. As expected, HFCD feeding led to significantly accelerated atherosclerosis, and after 7.0 weeks, a twofold increase in plaque area in the aortic arch was observed compared to chow-fed mice (Table 1; Figure 1A,B). A systematic review of the literature showed that, in the context of experimental atherosclerosis, a higher plaque burden is seen in young female hyperlipidaemic mice compared with their male counterparts [25]. Although we observed a trend towards females developing larger lesions than males after 7.0 weeks of HFCD, no significant difference was observed. No significant difference in lesion size between the HFCD- and chow-fed groups was observed at the 3.5-week time point (Table 1).

In line with the atherosclerosis data, HFCD-fed mice presented significantly higher levels of plasma cholesterol and triglycerides, especially after 7.0 weeks of diet feeding (Table 1). Although not significant within 7 weeks, HFCD presented a clear trend towards a faster weight gain than chow-fed mice (Supplementary Figure S1).

After confirming the atherosclerotic phenotype in our mice, we evaluated the effects of HFCD feeding on liver-related parameters. The liver-to-body ratio was significantly increased in *Apoe^−/−^* mice after 3.5 and 7.0 weeks of HFCD feeding (Table 1). These results were paralleled by significantly increased levels of plasma AST and ALT (Table 1). Additionally, hepatic accumulation of free iron was significantly increased in HFCD-fed mice compared to controls at the 7.0 weeks’ time point (Table 1).

Altogether, the previous results indicated that HFCD feeding of *Apoe^−/−^* mice promoted liver damage. Corroborating the latter affirmation, 7.0 weeks of HFCD feeding increased the hepatic levels of TNF and CCL2 (Table 1). Interestingly, the hepatic levels of IL-10 followed a different pattern, and substantially lower levels of the cytokine were seen after 7.0 weeks, which was independent of the diet (Table 1).

Considering that atherosclerosis and the first signs of NASH were observed after 7.0 weeks of HFCD, we selected this time point for further analyses. At this time point, HFCD clearly promoted hepatocyte ballooning (Figure 1A,B) and increased the accumulation of collagen, as evidenced by picrosirius red staining (Figure 1A,B). Further analyses showed that HFCD-fed mice presented significantly higher levels of hepatic cholesterol and triglycerides (Table 1), which was followed by a close to significant increase in hepatic hydroxyproline (Table 1) and a significant increase in *Col1a1* mRNA levels, which encodes for the pro-alpha1 chain of type I collagen (Table 1).

### 2.2. Genetic Ablation of IDO1 in Apoe^−/−^ Mice Accelerates Vascular, but Not Liver, Disease

**Figure 2:**
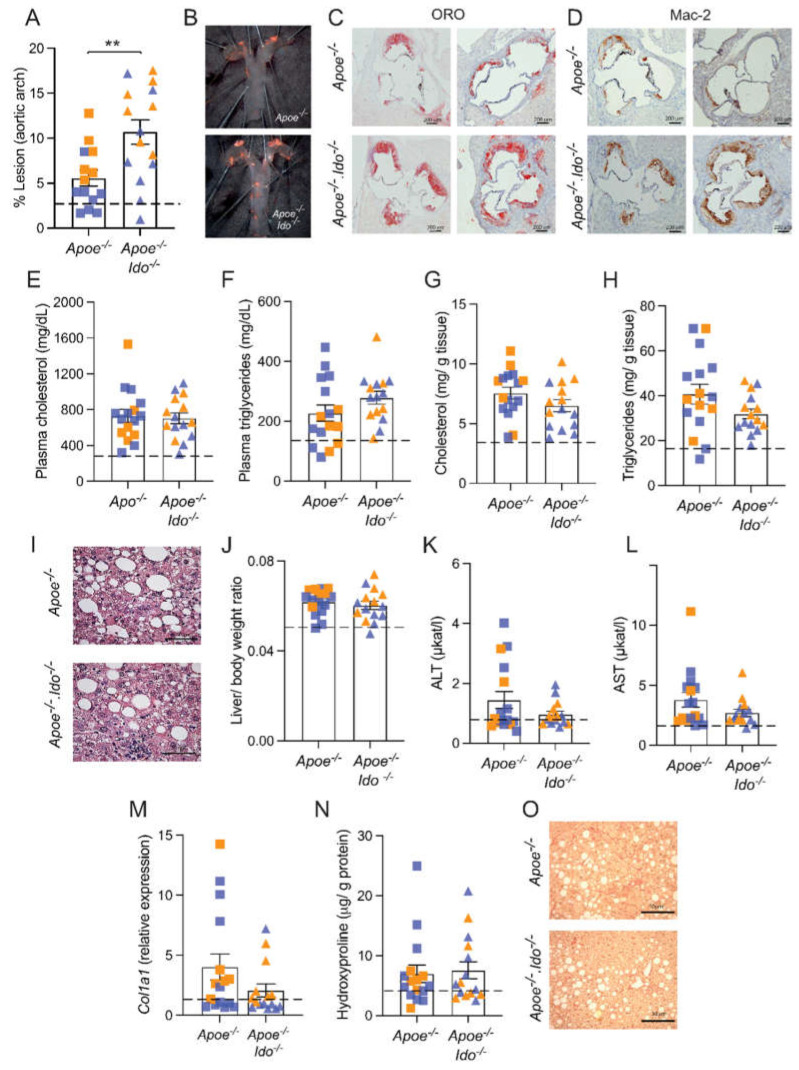
Effects of IDO1 genetic ablation on NASH and atherosclerosis development. *Apoe^−/−^* or *Apoe^−/−^Ido1^−/−^* mice were fed a high-fat cholesterol diet (HFCD) for 7 weeks; pooled data from two independent experiments are shown. (**A**) Quantification of *en face* Sudan IV-stained aortic arches (*n* = 14–15). (**B**) Representative pictures of the aortic arches. (**C**) Representative pictures of the atherosclerotic burden stained by ORO in aortic root sections of *Apoe^−/−^* or *Apoe^−/−^* *Ido1^−/−^* mice (*n* = 2/group). (**D**) Representative picture of Mac-2^+^ macrophage infiltration in the aortic roots of *Apoe^−/−^* or *Apoe^−/−^* *Ido1^−/−^* mice (*n* = 2/group). Panel (**E**) shows total cholesterol and (**F**) triglyceride levels in plasma (*n* = 15–16). (**G**,**H**) Total levels of cholesterol and triglycerides in *Apoe^−/−^* and *Apoe^−/−^Ido1^−/−^* mice livers (*n* = 15–16). (**I**) Representative pictures of H&E-stained liver sections. (**J**) Liver/body ratio (*n* = 15–16), (**K**,**L**) plasma ALT and AST levels (*n* = 15–16). (**M**) Relative hepatic collagen (*Col1a1*) mRNA expression (*n* = 15–16) and (**N**) hepatic hydroxyproline levels (*n* = 15–16). (**O**) Representative pictures of picrosirius red-stained liver sections. ** *p* < 0.01; differences were detected using the Mann–Whitney U test; dotted lines refer to baseline levels of *Apoe^−/−^* mice fed a chow diet for 7 weeks (Table 1); orange and blue colours are used to identify female and male mice, respectively. Bar = 50 µm.

Our data indicated that 7.0 weeks of HFCD feeding represents a suitable time point to study both atherosclerosis and NASH as concomitant diseases, and this protocol was selected to evaluate the role of IDO1 in disease. In line with our previous studies [9,10], ablation of IDO activity (*Apoe^−/−^Ido1^−/−^*) significantly increased atherosclerosis in the aortic arch compared to Apoe*^−/−^* mice (Figure 2A,B). Hence, *Apoe^−/−^Ido1^−/−^* mice also presented more lesions in the aortic root and increased Mac-2^+^ macrophage infiltration compared to *Apoe^−/−^* controls (Figure 2C,D).

Next, we evaluated whether IDO1 ablation would impact NASH-related parameters in our model. There was no difference in plasma cholesterol and triglycerides (Figure 2E,F) or bodyweight between groups (Supplementary Figure S2). In line with the plasma data, *Apoe^−/−^* *and Apoe^−/−^Ido1^−/−^* mice presented no difference in the hepatic accumulation of cholesterol and triglyceride levels (Figure 2G,H), and similar hepatocyte ballooning was observed between groups (Figure 2I). Further analyses showed that *Apoe^−/−^* and *Apoe^−/−^Ido1^−/−^* also did not differ in terms of the liver-to-body weight ratio (Figure 2J), plasma levels of ALT and AST (Figure 2K,L), hepatic *Col1a1* mRNA levels (Figure 2M), and hydroxyproline content (Figure 2N). Corroborating with the latter result, no difference in the picrosirius red staining of collagen was observed between groups (Figure 2O).

**Figure 3:**
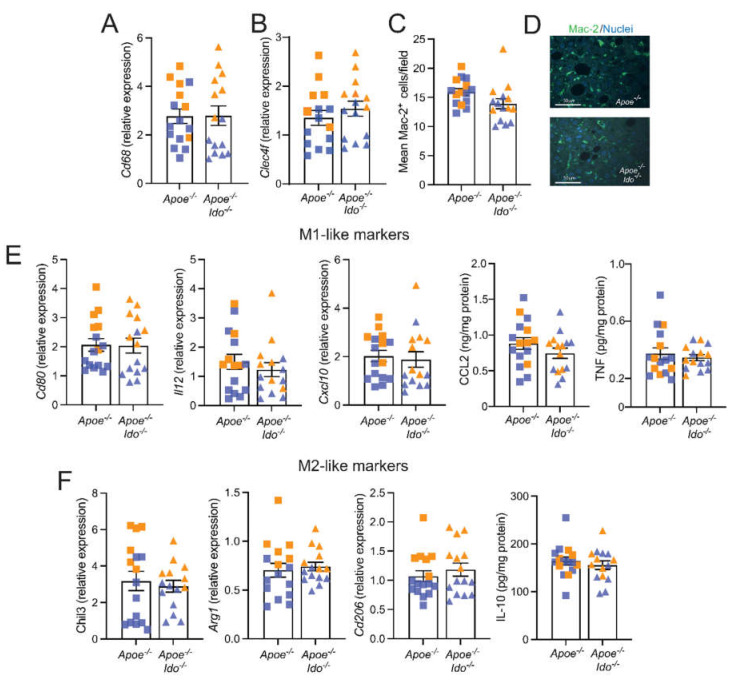
Effects of IDO1 genetic ablation on liver inflammation. *Apoe^−/−^* or *Apoe^−/−^Ido1^−/−^* mice were fed a high-fat cholesterol diet (HFCD) for 7 weeks; pooled data from two independent experiments are shown. (**A**) Relative *Cd68* mRNA expression (*n* = 15–16); (**B**) Relative *Clec4f* mRNA expression (*n* = 15); (**C**) Quantification of immunofluorescently stained Mac-2^+^ macrophages (*n* = 15); and (**D**) representative Mac-2^+^ fluorescence staining of liver from *Apoe^−/−^* or *Apoe^−/−^Ido1^−/−^*; Bar = 50 µm. (**E**) Relative hepatic mRNA expression of M1-like macrophage markers (*Cd80*, *Il12*, *and Cxcl10* mRNA, and CCL2 and TNF protein) (*n* = 15–16) and (**F**) M2-like macrophage markers (*Chil3*, *Arg1*, *and Cd206* mRNA, and IL-10 protein) (*n* = 15–16). Orange and blue colours are used to identify female and male mice, respectively; no differences between groups were detected using a Mann–Whitney U test.

IDO1 ablation is usually followed by increased inflammation in different disease models [26,27], including atherosclerosis [8,9,10]. Immunofluorescence and macrophage-related transcript analyses revealed that *Apoe^−/−^* and *Apoe^−/−^Ido^−/−^* mice presented no differences in the hepatic infiltration of macrophages and the mRNA levels for the Kupfer cell marker *Clec4f* (Figure 3A–D) and no clear shift towards M1- or M2-like macrophage polarization patterns (Figure 3E,F). Of note, M1 and M2 terminologies are an oversimplification of a vast repertoire of phenotypes that can develop within an inflamed tissue, including atherosclerosis [28].

### 2.3. Apoe^−/−^Ido1^−/−^ Mice Presented Intact Hepatic Trp Degradation Rates despite Reduced Systemic and Aortic Trp Degradation Rates

**Figure 4:**
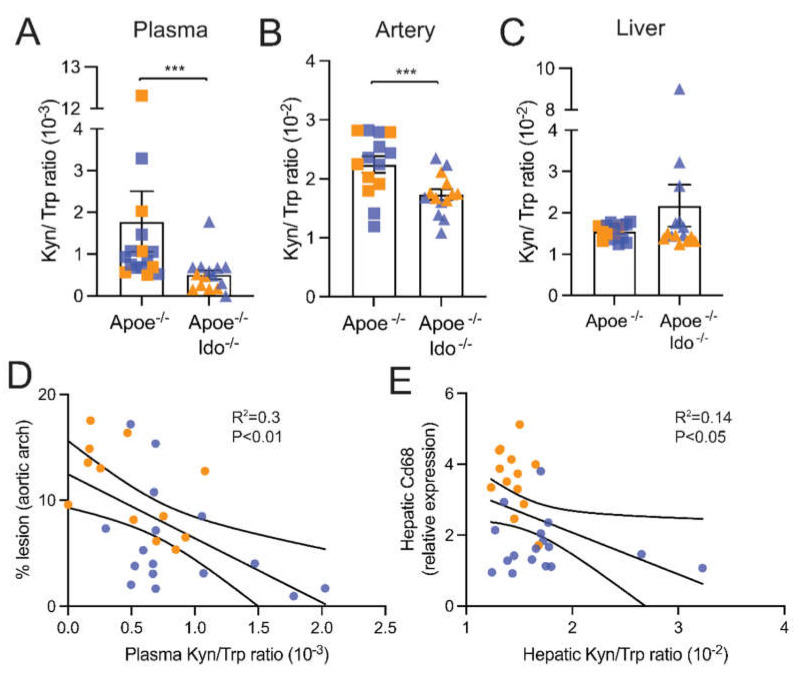
Systemic and local tryptophan degradation rates. *Apoe^−/−^* or *Apoe^−/−^* *Ido1^−/−^* *mice* were fed a high-fat cholesterol diet (HFCD) for 7 weeks; pooled data from two independent experiments are shown. The L-kynrenine to Trp ratio (Kyn/Trp) in (**A**) plasma (*n* = 15–16), (**B**) artery homogenate (*n* = 14), and (**C**) liver homogenate (*n* = 15–16), was estimated using specific ELISA kits, as described in the methods. (**D**) shows the correlation between % lesion and Kyn/Trp ratio in the aorta. (**E**) shows the correlation between relative *Cd68* mRNA and the Kyn/Trp ratio in the liver. *** *p* < 0.001; Differences were detected using the Mann–Whitney U test. Correlations were determined using simple linear regression.

The Kyn/Trp is used as a surrogate marker of IDO1 activity and the degradation of Trp within the kynurenine pathway. As expected, the Kyn/Trp ratio was reduced in plasma and aortas from *Apoe^−/−^Ido1^−/−^* mice compared to *Apoe^−/−^* mice (Figure 4A,B). Unexpectedly, no difference in the hepatic Kyn/Trp ratio was observed between the groups (Figure 4C). In line with the fact that increased rates of Trp degradation are usually associated with decreased inflammation, we found that the aortic Kyn/Trp ratio was inversely correlated with the percentage of lesions in the aortic arch (Figure 4D). Despite no difference between groups on Mac-2^+^ macrophage numbers, the hepatic Kyn/Trp ratio was also inversely correlated with the macrophage marker CD68 (Figure 4E), suggesting that hepatic inflammation could be regulated by the degree of Trp degradation in the liver.

### 2.4. HFCD Increases Hepatic TDO2 Expression

**Figure 5:**
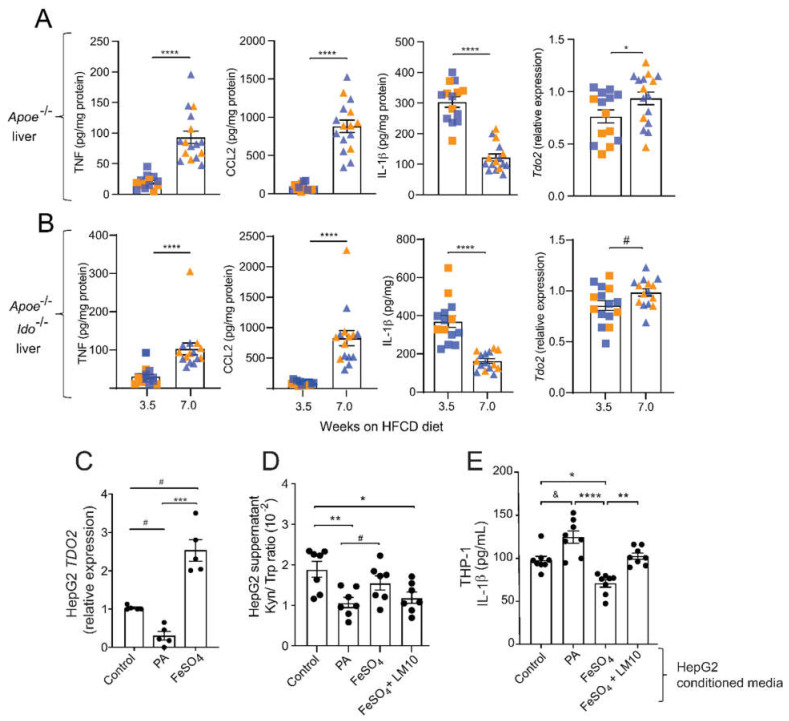
Fatty acids and iron regulate TDO2-dependent Trp degradation and consequences for THP-1 macrophage activation. (**A**,**B**) TNF, CCL2, and IL-1β protein levels and relative *Tdo2* mRNA expression in livers from *Apoe^−/−^* and *Apoe^−/−^Ido1^−/−^* mice fed a high-fat cholesterol diet (HFCD) for 3.5 and 7 weeks. (**A**,**B**) Pooled data from two independent experiments are shown; *n* = 14–15/group. © Relative expression of TDO2 mRNA in HepG2 cells treated with palmitic acid (PA, 500 μM) or iron (FeSO_4,_ 100 μM) (*n* = 5). (**D**) Kyn/ Trp ratio in the supernatants of HepG2 cells treated with PA (500 μM), FeSO_4,_ (100 μM), or FeSO_4_ + TDO2-inhibitor LM10 (0.62 μM) (*n* = 7). (**E**) IL-1β release from THP-1 macrophages pre-treated with conditioned media from HepG2 cells incubated with PA (500 μM), FeSO_4_ (100 μM), or FeSO_4_ + TDO2-inhibitor LM10 (0.62 μM); in addition, cells were stimulated with LPS (10 ng/mL, 4 h) and ATP (5 mM, 30 min) for activation of the inflammasome and IL-1β release (*n* = 8). ^&^ *p* < 0.06; ^#^ *p* < 0.08; * *p* < 0.05; ** *p* < 0.01; *** *p* < 0.001; **** *p* < 0.0001. (**A**) Differences were detected using the Mann–Whitney U test. (**B**,**C**) Differences were detected using a one-way ANOVA and Dunn’s post hoc test.

Although IDO1 has been implicated in the regulation of inflammation, another enzyme, tryptophan-2,3-dioxygenase (TDO2), is also involved in the first and rate-limiting step of the kynurenine pathway [29]. We observed a clear trend towards a temporal increase in the hepatic levels of *Tdo2* mRNA between 3.5 and 7.0 weeks of HFCD feeding in *Apoe^−/−^Ido1^−/−^* mice (Figure 5). Interestingly, a similar increase in *Tdo2* mRNA was observed in *Apoe^−/−^* (Figure 5). Although hepatic expression of TDO2 could be one explanation for the maintenance of Trp degradation and protection against the aggravation of liver disease in *Apoe^−/−^Ido1^−/−^*, the similar increase seen in the liver of *Apoe^−/−^* mice suggests that other mechanisms could influence Trp degradation in the presence of IDO1. Interestingly, aortic TDO2 protein levels were decreased in *Apoe^−/−^Ido1^−/−^* compared to *Apoe-/-* mice after 3.5 weeks of HFCD feeding, while no difference was observed between groups at the 7.0 weeks’ time point (Supplementary Figure S3).

We have shown that the kynurenine pathway metabolism can regulate inflammasome activation and IL-1β secretion by macrophages [30]. In line with these data, IL-1β levels have been found to be increased in the plasma of *Tdo2^−/−^* mice injected with LPS [31]. In our comorbidity model, *Apoe^−/−^ and Apoe^−/−^Ido1^−/−^* mice showed a time-dependent increase in the hepatic levels of TNF and CCL2, while a concomitant decrease in the hepatic levels of IL-1β was observed (Figure 5), suggesting that the latter could be regulated by TDO2.

An increased accumulation of lipids and free iron are well-known characteristics of NASH progression [32], which was also observed in our model (Table 1 and Figure 1). Interestingly, TDO2 is a tetrameric haemoprotein that requires Fe^2+^ for its full activation as other catalytic haemoproteins, and iron has been proposed to upregulate *TDO2* mRNA expression levels [33]. Considering all the previous, we tested whether excess palmitic acid (PA) or iron (FeSO_4_) could regulate the expression of *TDO2* in the liver hepatoma cell line HepG2, and whether TDO2-mediated Trp metabolism on hepatic cells could influence IL-1β secretion by macrophages.

Forty-eight hours of incubation of HepG2 cells with PA downregulated, while FeSO_4_ substantially increased, *TDO2* mRNA levels (Figure 5C). Analyses of the supernatant of these cultures showed a decrease in the Kyn/Trp ratio in the supernatants of HepG2 cells treated with PA, while no changes were observed in cells treated with FeSO_4_ (Figure 5D). Interestingly, the concomitant addition of the TDO2 inhibitor LM10 to HepG2 cells treated with FeSO_4_ showed a reduced Kyn/Trp ratio compared to the control (Figure 5D).

Next, we tested whether the regulation of *TDO2* expression on HepG2 cells, and reflected alterations in Kyn/Trp ratio, could, in a paracrine manner, influence the response of THP1-differentiated macrophages to secrete IL-1β in vitro. We observed that conditioned media from HepG2 cells cultured with PA, which reduced their *TDO2* expression and Kyn/Trp ratio, increased the secretion of IL-1β by THP1 macrophages (Figure 5E). Contrary to the effects of PA, the conditioned media from FeSO_4_-treated HepG2 cells, which upregulated *TDO2* and maintained an unchanged the Kyn/Trp ratio, significantly inhibited IL-1β secretion; these protective properties were lost when HepG2 cells concomitantly received FeSO_4_ and the TDO2 inhibitor LM10 (Figure 5E).

## 3. Discussion

NAFLD/NASH typically exists within the ‘‘milieu” of major diseases that play a central role in increasing the risk of CVD, including obesity, diabetes, and dyslipidaemia. Not surprisingly, myocardial infarction and stroke are highly prevalent in patients with metabolic liver disease [23]. Increasing our knowledge of the underlying mechanisms by which NAFLD/NASH accelerates atherosclerosis and increases cardiovascular risk can help improve the diagnosis and management of CVDs. In this study, we show that HFCD feeding promotes NASH and atherosclerosis in parallel in *Apoe^−/−^* mice, establishing a new viable dual comorbidity model. By feeding *Apoe^−/−^Ido1^−/−^* mice with HFCD_,_ we show that IDO1-dependent Trp metabolism plays a distinctive role in regulating vascular versus fatty liver disease.

There have been numerous attempts to generate animal models, especially murine models, that can recapitulate the aetiology, natural history, and/or progression that are inherent to atherosclerosis or NAFLD/NASH [34,35]. In this context, the two most common hypercholesterolaemic mouse strains used to study atherosclerosis, *Apoe^−/−^* and *Ldlr^−/−^*, have been evaluated regarding their susceptibility to developing NASH. Schierwagen et al., (2015) showed that 7 weeks of HFCD feeding led *Apoe^−/−^* mice to develop several features common to human NASH, including hepatic steatosis, inflammation, and a moderate degree of fibrosis [36]. Bieghs et al., (2012) showed that *Ldlr^−/−^* mice present increased sensitivity to hepatic inflammation, apoptosis, and fibrosis after 12 weeks of HFCD compared to the human *APOE2* knock-in mouse (*APOE2ki*) and C57BL/6 strains [37]. Despite the potential within these models, atherosclerosis has not been investigated in these studies.

To date, only a few studies have attempted to explore disease-modifying targets that could concomitantly influence NASH and CVD. Recently, van den Hoek et al., (2020) have shown that *Ldlr^−/−^. Leiden* mice develop NASH with progressive liver fibrosis, as well as atherosclerosis, upon 28 weeks of special high caloric diet feeding [38]. In our study, we established that HFCD feeding of *Apoe^−/−^* mice could also be a suitable strain for studying NASH and atherosclerosis simultaneously with a swift 7-week protocol. Thus, in addition to CVD, our mice presented all clinical signs that are characteristic of NASH, including liver steatosis, cytoskeletal damage (hepatocellular ballooning and increased levels of liver enzymes), inflammation, and a moderate degree of fibrosis, which, although not required for disease diagnosis, may indicate the aggravation of the disease state [39,40].

Inflammation is the major regulator of IDO1-dependent Trp metabolism in different cells and organs [41]. Increased IDO1 activity has been considered an important immune metabolic feedback mechanism regulating innate and adaptive immune cell responses [8]. Whether operating directly or indirectly, increased Trp metabolism through the kynurenine pathway has been linked with CVD because of its role in regulating vasculature [42], insulin resistance [30,43,44,45], or skewing of the gut microbiota [21]. Taking all previous knowledge into account, IDO1 emerged as an interesting target to be investigated in our dual model.

As we have previously shown using pharmacological and genetic approaches [9,10], IDO1 ablation increases vascular inflammation and accelerates atherosclerosis, which could now be reproduced using a HFCD. Unexpectedly, in the current study, we did not observe an acceleration of liver disease. In light of the fact that using a downstream metabolite of IDO in the kynurenine pathway, 3-hydroxyanthranilic acid (3-HAA), could regulate cholesterol synthesis as well as plasma and hepatic cholesterol levels [30], our new data might appear counterintuitive. While further research will be needed to fully understand the potential causes of these differences, some hypothetical lines of reasoning could be drawn.

We previously showed that 3-HAA mediated strong lipid-lowering effects in *Ldlr^−/−^* mice [30,46]. Hence, it was shown that genetic ablation of IDO in *Ldlr^−/−^* led to a significant increase in plasma lipids [47]. When using *Apoe^−/−^* mice, the pharmacological inhibition of IDO promoted only mild alterations to their lipoprotein profile, while four weeks of treatment with 3-HAA did not reverse the effects of IDO1 inhibition on lipids [46]. Interestingly, the original work from Cole et al., (2015) showed that *Apoe^−/−^Ido^−/−^* mice presented no overt alteration in plasma lipids under a chow diet [10], suggesting that the strain background could play a major role in how mice respond to variations in IDO1-mediated Trp metabolism. Considering that the kynurenine pathway has been implicated in the regulation of SREBP-2 [30], which, in addition to regulating cholesterol synthesis, also regulates LDLR expression, it seems plausible that the presence of LDL-receptor in the model could have implications to the degree of hepatic lipid accumulation [47].

As expected, we found that the Kyn/Trp ratio was decreased in the arteries and plasma from *Apoe^−/−^Ido1^−/−^* mice, compared to *Apoe^−/−^* mice. Surprisingly, the Kyn/Trp ratio in the liver of both groups was not different at the end of the experiment, suggesting that compensatory mechanisms might have been triggered in *Apoe^−/−^Ido1^−/−^* mice under HFCD or NASH that could maintain Trp degradation rates. In the context of human liver disease, it has been shown that high Kyn/Trp ratio is associated with greater liver fibrosis in the context of HIV and HCV infections, as well as in patients with acute decompensation and acute-on-chronic liver failure cirrhosis [48,49]. Considering that in the current work, after 7.0 weeks of HFCD, our model developed just early stages of liver disease and mild fibrosis, we can speculate that worsening of NASH could lead to altered kynurenine pathway metabolism, which needs to be validated in future studies. In line with the previous thought, it has been shown that kynurenine pathway activity was found to be normal in patients with compensated cirrhosis, and only changed with aggravation of the disease [49].

While IDO1 is thought to be an inducible enzyme triggered especially by proinflammatory factors such as interferon-γ (IFNγ), Trp can also be degraded by two other enzymes, the IDO1 paralogues IDO2 and TDO2. It has been suggested that TDO2 is constitutively expressed in the brain and in the liver. While some regulation redundancy/overlap between IDO1 and IDO2 expression has been suggested, it has been thought that TDO2 expression is mainly mediated by glucocorticoids and other hormones [29]. In our study, we observed that TDO2 is upregulated in the liver of *Apoe^−/−^Ido1^−/−^*, as well as *Apoe^−/−^* mice over time on diet, suggesting that alterations in lipids and/or inflammation, known to be induced in hyperlipidaemic mice over time [50], could regulate hepatic TDO2 regulation. Hence, the fact that TDO2 protein expression in the aortas do not follow the same pattern suggests that this enzyme plays a rather liver-specific role.

It has been shown in a murine model of liver fibrosis with CCL4 that hepatic *Tdo2* is upregulated in *Ido1^−/−^* mice [18]. In this study, the authors showed that *Tdo2* upregulation was associated with increased expression of the general control nonderepressive-2 kinase (GCN2), a key nutrient sensor that is also known to be regulated by changes in amino acid metabolism [51]. As mentioned earlier, TDO2 is a haemoprotein that requires Fe^2+^ for its full activation. Hence, it has been proposed that haem promotes the de novo synthesis of TDO2, which constitutes an important mechanism of regulation of Trp degradation by this enzyme [33]. Using HepG2 hepatoma cultures, we found that excess fatty acids significantly downregulated, while iron upregulated *TDO2* mRNA levels. Considering that lipid and iron accumulation are common features of NAFLD/NASH progression, our data suggest that these ‘nutrients’ could be involved in the transcription regulation of hepatic *TDO2*, regulation of Trp metabolism, as well as control of hepatic inflammation.

As previously mentioned, IDO1-mediated immunoregulatory mechanisms could be the consequence of Trp depletion or the production of bioactive metabolites. In this context, there are bulk data indicating that kynurenines can influence immune responses in a paracrine fashion, e.g., the overexpression of IDO1 by tumours increases the production of L-Kyn and 3-HAA that can signal to inhibit effector T-cell responses or promote Treg differentiation [52,53]; the latter two outcomes have been recognized as an important mechanism of immune escape by tumours. Using conditioned media from HepG2 cells that were treated with palmitic acid or iron, on THP-1 macrophages, indicated that the regulation of TDO2 expression and Trp hepatic catabolism, through the kynurenine pathway, could constitute an important mechanism of communication between hepatocytes and macrophages, and the development of liver inflammation, particularly driven by IL-1β.

Surprisingly for us, *Tdo2* expression increased over time, not only in the livers of *Apoe^−/−^ Ido1^−/−^* but also in *Apoe^−/−^* mice fed HFCD, which can express *Ido1*; however, the upregulation of *Tdo2* in the latter strain did not result in increased hepatic Trp degradation. These results raise two major thoughts: first, that TDO2-associated Trp metabolism and its potential influence on liver inflammation is independent of IDO1; and second, that in the context of NAFLD/NASH, hepatic Trp levels and metabolism is more complex than we anticipated. The uptake of Trp is thought to be driven essentially by the L-type neutral amino acid transporter 1 (*LAT1* or *Slc7a5*). In this context, it has been shown that LPS and TNF significantly reduce *LAT1* and Trp uptake in neuron-like cells [54], while IL-1β upregulates *LAT1* levels in fibroblast-like synoviocytes [55]. These findings suggest that LAT1 is an interesting candidate for future research involving Trp metabolism in the context of liver disease. Notably, the uptake of Trp can be regulated due to competition with other amino acids, and alterations in the levels and metabolism of amino acids, besides Trp, have been associated with NAFLD, e.g., the other aromatic amino acids tyrosine and phenylalanine, arginine, and branched-chain amino acids [56,57]. However, less is known about these other amino acids and their role in the regulation of immunometabolic responses, warranting further investigation.

In conclusion, despite the small size and short study design, we demonstrate that *Apoe^−/−^* mice fed HFCD for 7 weeks is a plausible model to study liver disease with atherosclerosis as a major comorbidity. Evaluation of the effects of *Ido1* genetic ablation revealed that this model may be used to better understand the dichotomies between vascular and hepatic inflammatory processes. A complete understanding of the role of IDO1 in the modulation of cardiovascular and liver disease as a comorbidity warrants further investigations. These could include the compensatory effects of other kynurenine pathway enzymes, and the potential crosstalk between hepatic and immune cells mediated by different Trp metabolites. A better understanding of these molecular processes could have implications for the design of high-precision therapies that can benefit both atherosclerosis and NAFLD/NASH.

## 4. Methods

### 4.1. Animal Model

The *Apoe^−/−^Ido1^−/−^* mouse strain was generated by crossing *Apoe^−/−^* mice with *Ido1^−/^*^−^ mice at the Kennedy Institute of Rheumatology, Oxford, UK [10]. The strain was transferred to the Center for Molecular Medicine at the Karolinska Institute in Stockholm and bred with *Apoe^−/−^* (B6.129P2-Apoe^tm1Unc/J^, strain code 622, JAX™, Charles River, The Netherlands) to generate *Apoe^−/−^* and *Apoe^−/−^Ido1^−/−^* littermate control mice that were used in the study; all mice were kept in specific pathogen-free (SPF) conditions with a 12-h light/dark cycle throughout the study. The model of concomitant development of atherosclerosis and NASH was achieved by adapting the protocol from Schierwagen et al. (2015) [36]. Briefly, 10-week-old male mice were fed normal chow or a high-fat, cholesterol-rich diet (HFCD) containing 42% kcal from fat, 43% kcal from carbohydrate, 15% kcal from protein, and 1.25% cholesterol (E15723-34, Sniff, Germany) ad libitum for 3.5 or 7 weeks. All animal experiments were performed in accordance with national guidelines and approved by the Stockholm Norra Regional Ethics Board (N28-15, approved on 26 March 2015), which conforms to the guidelines from Directive 2010/63/EU of the European Parliament on the protection of animals used for scientific purposes.

### 4.2. Atherosclerosis Burden Analyses

At the end of treatment, mice were euthanized with CO_2_. Blood was collected by cardiac puncture, and vascular perfusion was performed with sterile RNase-free PBS. After perfusion, the heart and aortic arch were dissected and preserved for lesion and immunohistochemistry analyses. We have previously shown that *Ido1* genetic and pharmacological ablation increases plaque burden in the aortic root [9,10]. In this study, aortic root sections obtained from cryopreserved hearts were used to obtain representative micrographs of plaque burden, which confirmed our previous publications. Lesion size was visualized on haematoxylin- and oil red O-stained sections as previously described [58]. Macrophage content in plaques was visualized using primary antibodies against Mac-2 (Cedarlane Laboratories, Burlington, ON, Canada) that were applied to acetone-fixed cryosections. Detection was performed using an ABC alkaline phosphatase kit (Vector Laboratories, Burlingame, CA, USA) as previously described [59]. En face lipid accumulation in the mouse aortic arch was determined using Sudan IV staining. Images were captured using a Leica DC480 camera connected to a Leica MZ6 stereomicroscope (Leica, Wetzlar, Germany). The lesion area was calculated using ImageJ software (NIH, Bethesda, MD, USA). Samples that were damaged during processing or analysis were excluded from the study. For the assessment of plaques, samples were coded, and the evaluation was performed by trained personnel who were blinded to the treatment groups.

### 4.3. Histological Analysis of Liver Disease Burden

The livers were dissected, and analogous samples were either snap-frozen or fixed in 4% phosphate-buffered formaldehyde for histopathology analyses. After fixation for 24–48 h, samples were dehydrated in a series of graded alcohols and embedded in paraffin wax. Serial sections of 5 μm were rehydrated and subjected to haematoxylin and eosin staining for morphological visualization of liver damage, and Picrosirius red (Fluka-Sigma Aldrich, Switzerland) to evaluate the extent of fibrosis. Hepatic macrophage content was evaluated using a primary antibody against Mac-2 (Cedarlane Laboratories, Burlington, Canada) that was detected using goat anti-rat IgG (DyLight^®^ 594) as the secondary antibody (Abcam, Cambridge, UK), and nuclei were stained with DAPI (Sigma Aldrich, St. Louis, MO, USA). All histological assessments were performed by a trained examiner who was blinded to the groups.

### 4.4. Biochemical Parameters in Liver and Blood

Analogous segments of snap-frozen livers were lysed in RIPA buffer using a TissueLyser II (Qiagen, Germantown, MD, USA). Hepatic hydroxyproline content was evaluated using a colorimetric assay kit (Sigma-Aldrich, St. Louis, MO, USA) according to the manufacturer’s instructions. Liver lipids were extracted from liver samples using the Folch method [60]. Briefly, lysates were homogenized in methanol, and lipids were extracted by chloroform separation (methanol: chloroform (1:2)). After drying, the extracts were redissolved in 1% Triton-100, and cholesterol and triglyceride contents were measured using enzymatic colorimetric kits (Randox Lab. Ltd. Crumlin, UK) according to the manufacturer’s instructions. Biochemical parameters (alanine aminotransferase (ALT) and aspartate aminotransferase (AST)) in blood were evaluated on a Samsung PT10V clinical chemistry analyser. Plasma cholesterol and triglycerides were measured using enzymatic colorimetric kits (Randox Lab. Ltd., Crumlin, UK) according to the manufacturer’s instructions. Hepatic free iron content was determined using a colorimetric assay kit (Sigma-Aldrich, St. Louis, MO, USA) following the manufacturer’s instructions.

### 4.5. Evaluation of Inflammatory Markers

In addition to immunohistological analyses, inflammation was evaluated in liver samples by qPCR. RNA was isolated from mouse livers using a RNeasy kit (Qiagen, Hilden, Germany). After approving the quality of the RNA on a NanoDrop (Thermo Scientific, Waltham, MA, USA), it was reverse transcribed with a High-Capacity RNA-to-cDNA™ Kit (Thermo Scientific, Waltham, MA, USA ) and amplified by real-time PCR using assay-on-demand primers and probes (*Il12*, *Cd80*, *Cxcl10*, *Chil3*, *Arg1*, *Cd206*, *Tdo2*, *TDO2*; all from Thermo Scientific, Waltham, MA, USA) in an ABI 7700 Sequence Detector (Applied Biosystems, Foster City, CA, USA). Hypoxanthine guanidine ribonucleosyl transferase (HPRT) was used as a housekeeping gene. Assay-on-demand primers and probes are provided in Supplementary Table S1. Data were analysed based on the relative expression method with the formula 2^−ΔΔCT^, where ΔΔCT = ΔCT (sample)–ΔCT (calibrator = average CT values of all samples within the control group) and ΔCT is the average CT of the housekeeping genes subtracted from the CT of the target gene. The levels of cytokines, including TNF- α, IL-1β, CCL2, and IL-10, were measured by ELISA according to the manufacturer’s instructions (all from R&D Systems, Minneapolis, MN, USA).

### 4.6. HepG2 Culture and Treatments

The human hepatoma cell line HepG2 was purchased from ATCC (VA, USA) and cultured as previously described [30]. Briefly, cells were maintained in low glucose (1 g/L) Dulbecco’s modified Eagle’s medium (DMEM, Sigma-Aldrich, St. Louis, MO, USA) supplemented with 10% foetal bovine serum (FBS), 2 mM L-glutamine, 100 units/mL penicillin, and 100 µg/mL streptomycin (all from Gibco, UK). At least 14 days prior to the experiment, the cells were passaged, and the medium was replaced with low glucose (1 g/L) DMEM supplemented with 2% AB+ human serum (Blodcentralen Karolinska Universitetssjukhuset, Sweden), 2 mM L-glutamine, 100 units/mL penicillin, and 100 µg/mL streptomycin. For mRNA analysis, cells were treated for 24 h with 500 μM palmitic acid (Sigma-Aldrich, St. Louis, MO, USA), 100 μM FeSO_4_ (Sigma Aldrich, St. Louis, MO, USA), 0.62 μM TDO inhibitor LM10 (Sigma-Aldrich, St. Louis, MO, USA) in different combinations, or vehicle as detailed in the figure legends. In parallel experiments, cells were washed after 24 h of treatment with palmitic acid or FeSO_4_, and the TDO inhibitor LM10 (all from Sigma-Aldrich, St. Louis, MO, USA), and new media was added. Supernatants of these cultures were saved after 48 h and used as conditional media in THP-1 cultures.

### 4.7. IL-1β Secretion by THP-1 Macrophages Treated with HepG2-Conditioned Media

The human monocytic cell line THP-1 was maintained in culture using RPMI 1640 (Invitrogen, MA, USA) culture medium containing 10% heat-inactivated FBS (Gibco, UK) supplemented with 2 mM L-glutamine, 100 units/mL penicillin, and 100 µg/mL streptomycin (all from Gibco, UK). THP-1 monocytes were then differentiated into macrophages by 24 h incubation with 100 ng/mL phorbol 12-myristate 13-acetate (PMA; Sigma-Aldrich, St. Louis, MO, USA), followed by 24 h incubation with conditioned media from HepG2 cells subjected to different treatments and upon stimulation with 10 ng/mL LPS (Sigma-Aldrich, St. Louis, MO, USA) for 4 h. After 4 h of incubation with LPS, cells were treated with 5 μM ATP for inflammasome activation and the release of IL-1β was measured by ELISA as previously described.

### 4.8. Kyn/Trp Ratio

The L-Kyn/Trp ratio, determined by ELISA (ImmuSmol, Bordeaux France), was used as a surrogate marker of IDO1-TDO2 activity in the aorta, liver, and plasma from 7-week HFCD-fed *Apoe^−/−^* and *Apoe^−/−^Ido1^−/−^* mice.

### 4.9. Statistical Analysis

The results are presented as the mean ± SEM if not otherwise stated. The Mann–Whitney U-test was used for comparisons between two groups, and Kruskal–Wallis ANOVA with Dunn’s post-test was used for comparisons between more than two groups. Correlations were calculated using simple linear regression analysis. *p* values < 0.05 were considered significant.

## Figures and Tables

**Table 1:** Characterization of HFCD-induced vascular and liver disease as comorbidities.

		*Apoe^−/−^*			
		Chow (^c^)		HFCD (^d^)	
		**3.5 Weeks** **(*n* = 10–12)**	**7.0 Weeks** **(*n* = 10–12)**	**3.5 Weeks** **(*n* = 11–14)**	**7.0 Weeks** **(*n* = 14–16)**
Aorta	% Lesion (aortic arch)	0.99 ± 0.29	2.88 ± 0.85 ^#3.5c^	0.48 ± 0.22	5.56 ± 0.89 ****^3.5d^
Plasma	Cholesterol (mg/dL)	381.9 ± 55.6	370.6 ± 38.2	739.8 ± 64.2 ***^3.5c^	732.6 ± 73.8 ***^7.0c^
	Triglycerides (mg/dL)	160.4 ± 22.2	144.6 ± 13.5	254.2 ± 43.9	226.9 ± 27.4 ***^7.0c^
	ALT (μkatl/L)	0.46 ± 0.04	0.62 ± 0.09	1.79 ± 0.44 ***^3.5c^	1.45 ± 0.29 ***^7.0c^
	AST (μkatl/L)	1.72 ± 0.17	1.92 ± 0.17	3.25 ± 0.62 *^3.5c^	3.79 ± 0.61 ***^7.0c^
Liver	Liver/Body weight (mg/g)	0.052 ± 0.002	0.050 ± 0.002	0.065 ± 0.002 ***^3.5c^	0.062 ± 0.001 ***
	Iron (ng/μL)	4.01 ± 0.60	3.54 ± 0.16	4.60 ± 0.22	5.13 ± 0.37 *^7.0^
	TNF (pg/mg tissue)	31.62 ± 2.77	38.72 ± 3.08	19.76 ± 2.82 **^3.5c^	93.19 ± 10.18 ***^7.0c;^ ***^3.5d^
	CCL2 (pg/mg tissue)	69.7 ± 10.7	200.3 ± 36.2	78.21 ± 12.4	880.6 ± 81.4 ****^7.0c;^ ****^3.5d^
	IL-10 (pg/mg tissue)	476.2 ± 44.4	28.07 ± 3.26 ****^3.5c^	1594 ± 145.4	164.1 ± 8.478 ****^3.5d^
	Cholesterol (mg/mg tissue)	—	3.42 ± 0.39	—	7.556 ± 0.49 ****^7.0c^
	Triglycerides (mg/mg tissue)	—	18.45 ± 2.53	—	40.74 ± 4.41 ****^7.0c^
	Hydroxyproline (μg/mg tissue)	—	4.05 ± 0.59	—	6.97 ± 1.47
	*Col1a1* (relative expression)	—	1.09± 0.134	—	4.03 ± 1.08 *^7.0c^

^#^) *p* < 0.07; *) *p* < 0.05; **) *p* < 0.01; ***) *p* < 0.001; ***) *p* < 0.0001; ^3.5c or d^ and ^7.0c or d^ indicates the made comparison; (c) chow; (d) HFCD.

## Data Availability

Available on request from the corresponding author.

## Supplementary Materials

The following supporting information can be downloaded at: https://www.mdpi.com/article/10.3390/ijms23095203/s1.
