# Supplementary material for: Genetic Deficiency of Indoleamine 2,3-dioxygenase Aggravates Vascular but Not Liver Disease in a Nonalcoholic Steatohepatitis and Atherosclerosis Comorbidity Model
Source: Int J Mol Sci. 2022 May 6;23(9):5203. doi: 10.3390/ijms23095203 (PMC9099704; doi:10.3390/ijms23095203)
Supplement: Supplementary file 1 [file ijms-23-05203-s001.zip › ijms-1608353-supplementary.pdf]

Genetic deficiency of indoleamine 2,3-dioxygenase aggravates vascular but not liver disease in a nonalcoholic steatohepatitis and atherosclerosis comorbidity model

Arora A[1,2], Tripodi GL[1,3*], Kareinen I[1], Berg M[1], Forteza MJ[1], Gisterå A[1], Nielsen SDG[2], Casagrande FB[3], Martins JO[3], Abdalla DSP[3], Cole JE[4], Monaco C[4], Ketelhuth DFJ[1, 2*]

SUPPLEMENTARY MATERIAL

Supplementary Table S1. List of TaqMan® Probes used for Real Time qRT-PCR.

Gene	Assay ID
Il12a	Mm00434169_m1
Cd80	Mm00711660_m1
Cxcl10	Mm00445235_m1
Tdo2	Mm00451269_m1
Chil3	Mm00657889_mH
Arg1	Mm00475988_m1
Cd206	Mm01329362_m1
TDO2	Hs01045944_m1

SUPPLEMENTARY FIGURES

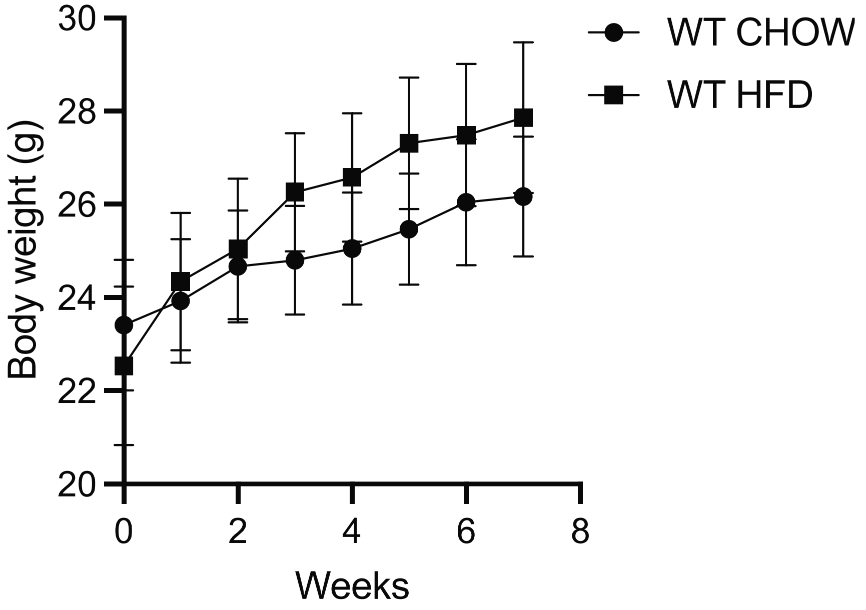

Supplementary Figure S1. Effect of high-fat cholesterol diet (HFCD) on body weight of *Apoe*$^{-/-}$ mice. Mice were fed chow or HFCD and their body weight was measured weekly for 7 weeks. The graph shows changes in the body weight over a period of 7 weeks. Values represent mean ± SEM (n= 7-8).

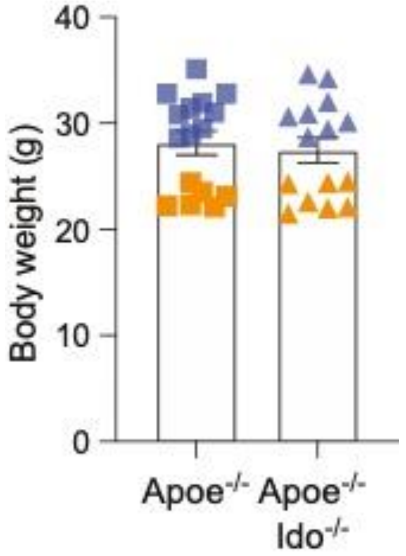

Supplementary Figure S2. Effect of IDO1 genetic ablation on HFCD- induced body weight gain in *Apoe*$^{-/-}$ mice. *Apoe*$^{-/-}$ or *ApoE*$^{-/-}$ *Ido1*$^{-/-}$ mice were fed HFCD and their body weight was measured at the end of 7 weeks. Values represent mean ± SEM (n=15-16). Pooled data from two independent experiments is shown. Orange and blue colours represent female and male mice, respectively.

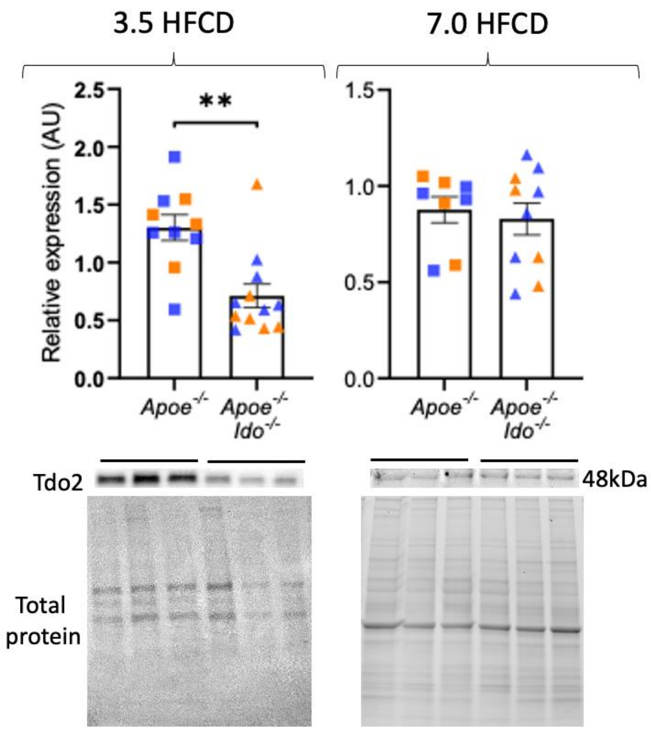

Supplementary Figure S3. Tdo2 protein expression in the arteries from HFCD fed mice. *Apoe*$^{-/-}$ and *ApoE*$^{-/-}$ *Ido1*$^{-/-}$ mice were fed HFCD for 3.5 and 7.0 weeks. Panels show Western blotting of Tdo2 in the protein extracts of arteries from both groups at the different time points (n = 8-12). Bottom panels show representative blots. Total protein staining was used as a loading control. Band intensity was measured and plotted normalized to the membrane background and total protein staining. Pooled data from two independent experiments is shown. Orange and blue colours represent female and male mice, respectively. **) P<0.01.
